# Transcriptomic Study of Mechanism Driving Conjunctival Fibrosis Following Glaucoma Filtration Surgery

**DOI:** 10.3390/biomedicines14081718

**Published:** 2026-07-31

**Authors:** Zoe Pasvanis, Antony Boynes, Roy C. K. Kong, Elsa C. Chan, Raymond C. B. Wong, Jennifer Fan Gaskin

**Affiliations:** 1 Centre for Eye Research Australia, East Melbourne, VIC 3002, Australia; zoepas2@gmail.com (Z.P.); roy.kong@unimelb.edu.au (R.C.K.K.); elsa.chan@unimelb.edu.au (E.C.C.); 2 Ophthalmology, Department of Surgery, University of Melbourne, Parkville, VIC 3010, Australia; 3 Department of Medicine, St. Vincent’s Hospital, University of Melbourne, Parkville, VIC 3010, Australia; 4 Glaucoma Research and Investigation Unit, Royal Victorian Eye and Ear Hospital, East Melbourne, VIC 3002, Australia

**Keywords:** glaucoma, transcriptomic, human tenon fibroblast, myofibroblast differentiation, fibrosis, TGFβ signalling

## Abstract

**Background**: Glaucoma filtration surgery (GFS) is performed to slow down disease progression in glaucoma, a leading cause of irreversible blindness worldwide. Following surgery, pathological wound healing may lead to conjunctival fibrosis and filtering failure. Myofibroblast transition of human Tenon’s fibroblast (HTF) is responsible for postoperative conjunctival scarring, with TGFβ signalling playing a key role in this process. However, the detailed molecular mechanism underlying myofibroblast transition in HTF remain understudied. **Methods**: In this study, we performed transcriptomic analysis to delineate the TGFβ1-induced changes in the transcriptome of HTF. HTF was isolated from three patients following GFS and treated with TGFβ1 for 5 days, followed by RNA-seq for transcriptomic profiling. **Results**: Our results identified 3362 differentially expressed genes (DEGs) in HTF following TGFβ1 treatment, of which 1532 were upregulated and 1820 were downregulated. Using gene ontology analysis, we identified signalling pathways associated with the pathogenesis of conjunctival fibrosis, including pathways involved in myofibroblast differentiation, TGFβ-signalling, collagen and extracellular matrix organisation, epithelial to mesenchymal transition, and cell cycle regulation. This study provided detailed characterisation of the transition from HTF to myofibroblast, and highlighted key genes that are upregulated (*LDLRAD4*, *CDKN2B*, *FZD8*, *MYOZ1*) and downregulated (*SOD3*, *LTBP4* and *RCAN2*) in this myofibroblast transition process. **Conclusions**: Overall, this study provided insights into the transcriptional landscape of HTFs and myofibroblast differentiation, which has important implication to understanding the pathophysiology of conjunctival scarring and development of new therapeutic agents.

## 1. Introduction

Glaucoma filtration surgery (GFS) is the gold-standard surgical procedure performed to lower intraocular pressure (IOP) in glaucomatous eyes. GFS allows for a gradual egression of aqueous humor from the anterior chamber of the eye into a surgically created subconjunctival space, resulting in the formation of a filtering bleb. As a result of surgical intervention, the body’s natural wound healing response is activated. In some cases, the magnitude of this response can be excessive, such that pathological wound healing is observed. This can lead to the postoperative complication of conjunctival fibrosis and surgical failure. Despite the use of adjunctive antimetabolite agents to control scarring, the operation still has a 50% failure rate over 5 years [1]. Frequently used antimetabolite agents such as Mitomycin C can result in toxicity of surrounding tissue, long-term wound breakdown and predispose the eye to severe infections that lead to blindness and even loss of the eye [2]. Therefore, there is an unmet need to further develop anti-scarring strategies.

One of the key effector cells responsible for postoperative conjunctival scarring is the human Tenon’s fibroblast (HTF). In an environment of injury and inflammation, as can be produced by GFS, HTFs are activated via transforming growth factor-beta (TGFβ) signalling [2]. As a result, HTFs change functionally and phenotypically into myofibroblasts, which are specialised contractile fibroblasts that produce excess extracellular matrix (ECM) components such as collagens and fibronectin [3]. While myofibroblasts are a vital component of physiological wound healing, an abundant accumulation at the surgical site is one of the major causes of surgical failure [2]. Interestingly Jeon et al. have recently shown that remodelling myofibroblasts to a fibroblast-like phenotype reduces fibrosis in a feline model of established corneal scarring [4]; therefore, targeting myofibroblasts by manipulating phenotypic changes may well be an effective way to limit fibrosis in post-GFS scarring.

Recent advances in RNA sequencing (RNA-seq) technologies have enabled comprehensive global profiling of gene expression, allowing unbiased characterisation of molecular mechanism in cellular processes. Since there has been no study investigating the molecular mechanisms of fibroblast to myofibroblast transition by HTF, the aim of the study is to establish the transcriptomic profiles of TGFβ1-induced conversion of HTF to myofibroblasts using RNA-seq.

## 2. Materials and Methods

### 2.1. HTF Isolation

**Figure 1:**
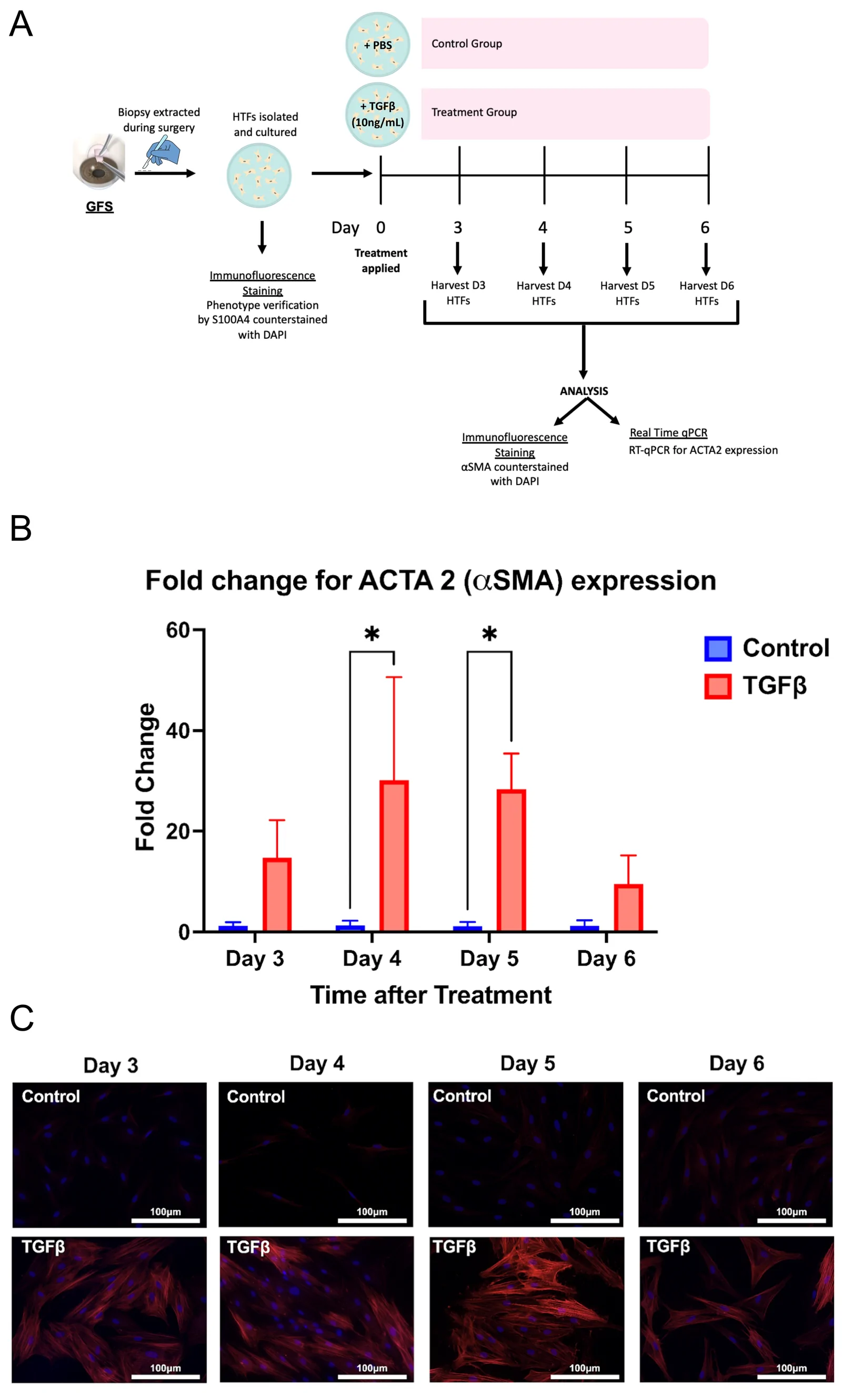
**Assessment of myofibroblast activation time-point following TGFβ stimulation.** (**A**) Preparation and treatment schedule of in vitro primary human Tenon’s fibroblasts for myofibroblast activation time-point determination (n = 3). (**B**) Fold change in expression of gene ACTA2 (encoding αSMA) in HTFs over 3–6 days following control and TGFβ treatment, n = 3. * Indicates significance (*p* < 0.05, two-way ANOVA). (**C**) HTF cell morphology 3–6 days following control and TGFβ treatment. αSMA stress fibres of myofibroblasts (red) identified by immunofluorescence microscopy (magnification 20×) with use of anti-αSMA and fluorophore-labelled secondary antibody. Nuclei counterstained with DAPI (blue).

**Figure 2:**
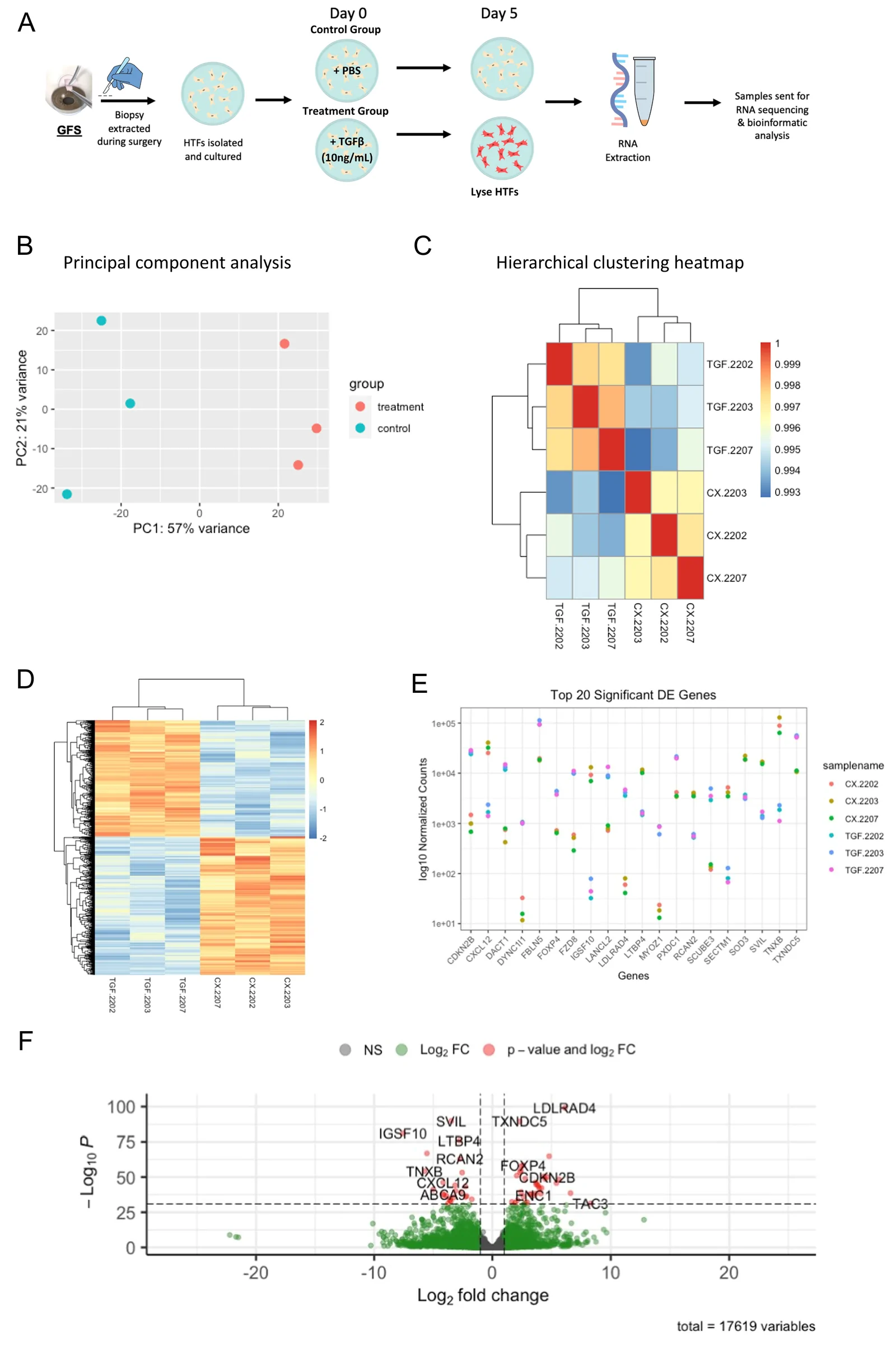
**Transcriptomic analysis of HTF following TGF β1 treatment.** (**A**) Preparation and treatment schedule of in vitro primary human Tenon’s fibroblasts for RNA sequencing (n = 3). (**B**) Principal component analysis (PCA) plot displaying 6 samples along PC1 and PC2, which describe 57% and 21% of the variability within the expression dataset respectively. PCA was applied to data following rlog transformation of normalised counts using DESeq2. Treatment group specified by symbol colour: blue = untreated control HTFs, red = TGFβ1-treated HTFs. (**C**) Hierarchical clustering map displaying correlation of gene expression for all pairwise combinations of samples in control (CX2202, CX2203, CX2207) and TGFβ1-treated groups (TGF2202, TGF2203, TGF2207). (**D**) Hierarchical clustering DEG heatmap by sample and transcripts on all significant genes using normalised counts. (**E**) Top 20 DEGs by adjusted *p* values of 0.05 inclusive of up- and downregulated genes ordered alphabetically. (**F**) Volcano plot showing DEG in HTF following TGFβ1 treatmen. Red dots on the right side of the plot indicate genes with significantly upregulated expression, while red dots on the left side of the plot indicate genes with significantly downregulated expression.

Isolation of HTFs was conducted in compliance with guidelines approved by our institutional ethics committee (The Royal Victorian Eye and Ear Hospital Human Research Ethics Committee project no. 16/1294H/21, approval date: 1 October 2016). Following explanation of the nature of the study, informed consent was obtained from patients. Explanted subconjunctival Tenon’s capsules (1 mm in length) were collected from patients (n = 3; 68-year-old Sri Lankan female, 68-year-old Turkish male and 80-year-old Greek female) during GFS and handled in accordance with the tenets of the Declaration of Helsinki. The tissue was attached to the bottom of a 35 mm cell culture dish (Thermo Fisher Scientific, Waltham, MA, USA) using a sterile coverslip and incubated in Dulbecco’s Modified Eagle Medium (DMEM; Sigma-Aldrich, St. Louis, MO, USA) containing Penicillin–Streptomycin–Glutamine (Thermo Fisher Scientific) and 10% fetal calf serum (FCS; Cytiva, Marlborough, MA, USA). Upon reaching confluence, the fibroblast monolayers were propagated, passaged, and subcultured in 75-cm^2^ tissue culture flasks. Subsequently, HTFs from the 3 patients were used for time-point assessment of TGFβ-induced myofibroblast activation (Figure 1A) and RNA-seq (Figure 2A).

### 2.2. Cell Culture

HTFs were maintained in DMEM (Sigma-Aldrich) containing Gibco Penicillin–Streptomycin–Glutamine (Thermo Fisher Scientific) and 10% fetal calf serum (FCS; Cytiva, Marlborough, MA, USA) at 37 °C with 5% CO_2_ in 50 mL and 160 mL cell culture flasks (Greiner Bio-One, Kremsmünster, Austria). HTFs were passaged when flasks achieved 80% confluency by dissociating monolayers with a Trypsin-EDTA solution (0.25%; Sigma-Aldrich). Fibroblasts obtained on passages 6 through 12, after initial cultures were established, were used. Phenotype verification of the cultured HTFs was achieved with the use of anti-fibroblast-specific protein 1 (S100A4) antibody (1:100; ABF32; Merck Millipore, Bayswater, VIC, Australia, Supplementary Figure S1). HTFs (approx. 40,000 cells/well) were seeded onto coverslips in 6-well Nunc cell culture plates (Thermo Fisher Scientific). Then HTFs were serum-starved in DMEM with 1% FCS overnight for 16–18 h prior to treatment. HTFs from the same patient were treated with TGFβ1 (10 ng/mL, as determined by us previously [5] or without TGFβ1 (control group). Following treatment, HTFs were fixed with 4% paraformaldehyde (PFA; P6148; Sigma-Aldrich, St. Louis, MO, USA) over a range of time-points (from days 3–14), or harvested for RNA-seq (day 5).

### 2.3. Immunocytochemical/Immunofluorescence Staining

Following fixation, HTFs were rinsed with Dulbecco’s Phosphate-Buffered Saline (DPBS; Thermo Fisher Scientific) and permeabilised with 0.1% Triton X-100 (Sigma-Aldrich) for 5–10 min and rinsed again with PBS. In order to prevent non-specific binding of the primary antibody, a blocking solution, UltraVision Protein Block (TA-125-PBQ; Thermo Fisher Scientific), was applied to coverslips for 30 min. HTFs on coverslips were then left to incubate overnight at 4 °C with the primary anti-’α-SMA antibody (M0851; Dako, CA, USA). Following an incubation period, HTFs on coverslips were rinsed thoroughly with PBS and were treated with a fluorophore-labelled secondary antibody, Alexa Fluor 568 goat anti-mouse IgG (H + L) (A11004; Thermo Fisher Scientific), for 1 h in the dark at room temperature (22 °C), then rinsed again with PBS. Nuclear counterstaining was conducted with the application of 4′,6-diamidino-2-phenylindole (DAPI) (1:1000; ab228549; Abcam, Cambridge, UK) for 10 min in the dark at room temperature. Finally, coverslips were mounted onto microscope slides with DPX mounting media (20242; Labworks, Knox City Centre, VIC, Australia). All slides were observed at 20× magnification using a fluorescence microscope (Axio Scope.A1 with Axiocam 105 colour; Zeiss, Oberkochen, Germany) and analysed with corresponding software (Zen 3.1 blue edition, Zeiss).

### 2.4. Real-Time qPCR

RNA extraction was performed using an illustra RNAspin Mini Kit (GE Healthcare Life Sciences, Marlborough, MA, USA), following the manufacturer’s instructions. RNA concentration and quality were measured using SimpliNano (GE Healthcare Life Sciences). cDNA was synthesised by reverse transcription of RNA. RT-qPCR was performed using TaqMan Fast Advanced Master Mix (4444554; Thermo Fisher Scientific) and Taqman gene expression assay probe for *ACTA2* (Hs00426835_g1) and the housekeeping gene 18s rRNA control mix (4318839; Thermo Fisher Scientific). RT-qPCR was performed on a QuantStudio™ 7 Flex Real-Time PCR System (Thermo Fisher Scientific), following the manufacturer’s instructions. The delta–delta Ct (ΔΔCT) method was used to calculate and compare relative mRNA levels to control. Data were expressed as mean ± standard deviation (SD) and were analysed with two-way analysis of variance (ANOVA) followed by post hoc Šídák’s multiple comparisons test. A value of *p* < 0.05 was regarded as statistically significant.

### 2.5. RNA Sequencing

RNA was extracted using the Illustra RNAspin Mini Kit (GE Healthcare Life Sciences) according to the manufacturer’s instructions. RNA quality was checked by a bioanalyzer, followed by library construction using the TruSeq Stranded mRNA kit (Illumina, San Diego, CA, USA), and sequenced using Novaseq 6000 (Illumina) with 100 bp single-end sequencing, at a depth of ~20 million reads per sample (Australian Genome Research Facility, Melbourne, Australia).

### 2.6. Bioinformatic Analysis

Following the abundance estimates of transcripts generated by *Salmon* v1.8, the pseudocounts were mapped to the GRCh38 genome assembly using the *tximport* v1.22.0 package. The gene count matrix was inputted as an DESeq2Dataset object using the *DESeqDataSetFromTximport* function, and then the DESeq2Dataset object was normalised using the *counts* function to make fair gene expression comparisons between samples [6]. The normalised dataset was analysed with the *DESeq2* v1.34.0 R package using rlog transformation. The sample-level quality assurance and distribution bias was measured using principal component analysis (PCA) while the gene-level QC was performed using hierarchical clustering. For differential expression analysis, the significant differentially expressed genes were determined using the *filter* function with adjusted *p* value of <0.05 and fold change > 2.0. Gene Ontology (GO) enrichment analysis was performed to investigate relationships between significantly expressed genes and their cellular component, biological process, and molecular function. Only terms with a *p*-value < 0.01 were considered significant. Network topology analysis was performed using Enrichr with the top 50 upregulated DEGs [7]. Gene set enrichment analysis was performed with GSEA 4.3.2 [8] and MSigDB 2023.1 [9], where a false-discovery rate of q-value < 0.25 and a *p*-value < 0.01 were considered significant. Further GSEA (gene set enrichment analysis) and pathway analysis were conducted using msigdbr [10] and clusterProfiler packages [11].

## 3. Results

### 3.1. Transition of HTF to Myofibroblast

Using primary HTFs, we first performed time-point analysis to determine the peak expression of the marker of myofibroblasts, alpha-smooth muscle actin (αSMA). We treated HTFs, derived from three GFS patients, with TGFβ1 for 3–6 days (Figure 1A). Following real-time qPCR, expression of *ACTA2* in TGFβ groups was significantly increased when compared to control at day 4 and day 5 (Figure 1B). In addition, TGFβ1 treatment induced morphological change of HTFs into myofibroblasts (Figure 1C, Supplementary Figure S2), as indicated by αSMA expression in collagen fibres 3 days post-treatment, with the highest expression of αSMA detected 5 days post-treatment.

### 3.2. RNA-Seq Analysis of Myofibroblast Transition

Since the peak phenotypic changes of fibroblasts to myofibroblasts occurred at day 5 following treatment with TGFβ1, we performed RNA-seq on HTFs from the control group (CX2202, CX2203, CX2207) and TGFβ1 treatment group (TGF2202, TGF2203, TGF2207) (Figure 2A). Principal component analysis demonstrated that biological repeats of the TGFβ1-treated group or control group cluster together, supporting similarities between biological repeats (Figure 2B). Similarly, hierarchical clustering showed a correlation of gene expression for all pairwise combinations of biological repeats of the treatment group (Figure 2C).

A total of 35,691 genes were identified in all samples of RNA-seq on primary HTFs. Following DESeq2 analysis and hierarchical clustering, there were 3362 DEGs identified, of which 1532 were upregulated and 1820 were downregulated in HTF following TGFβ1 treatment (with an adjusted *p* value of <0.05, Figure 2D). The top 20 DEGs between the two groups are visualised in both a dotplot (Figure 2E) and volcano plot (Figure 2F). The normalised dot plot visualises the differential expression of the top 20 genes in our gene set by relative position (determined by log10 normalised counts) of the TGFβ1 treatment group sample in comparison to the control group sample. Our results identified key genes that are upregulated (*LDLRAD4*, *CDKN2B*, *FZD8*, *MYOZ1*) and downregulated (*SOD3*, *LTBP4* and *RCAN2*) in this TGFβ1-induced myofibroblast transition process. Also, our results highlighted upregulation of known mediators of myofibroblast differentiation, such as *NOX4* (Log2FC = 6.62, Supplementary Data S1).

### 3.3. TGFβ1 Promotes Cell Cycle Arrest in HTFs

**Figure 3:**
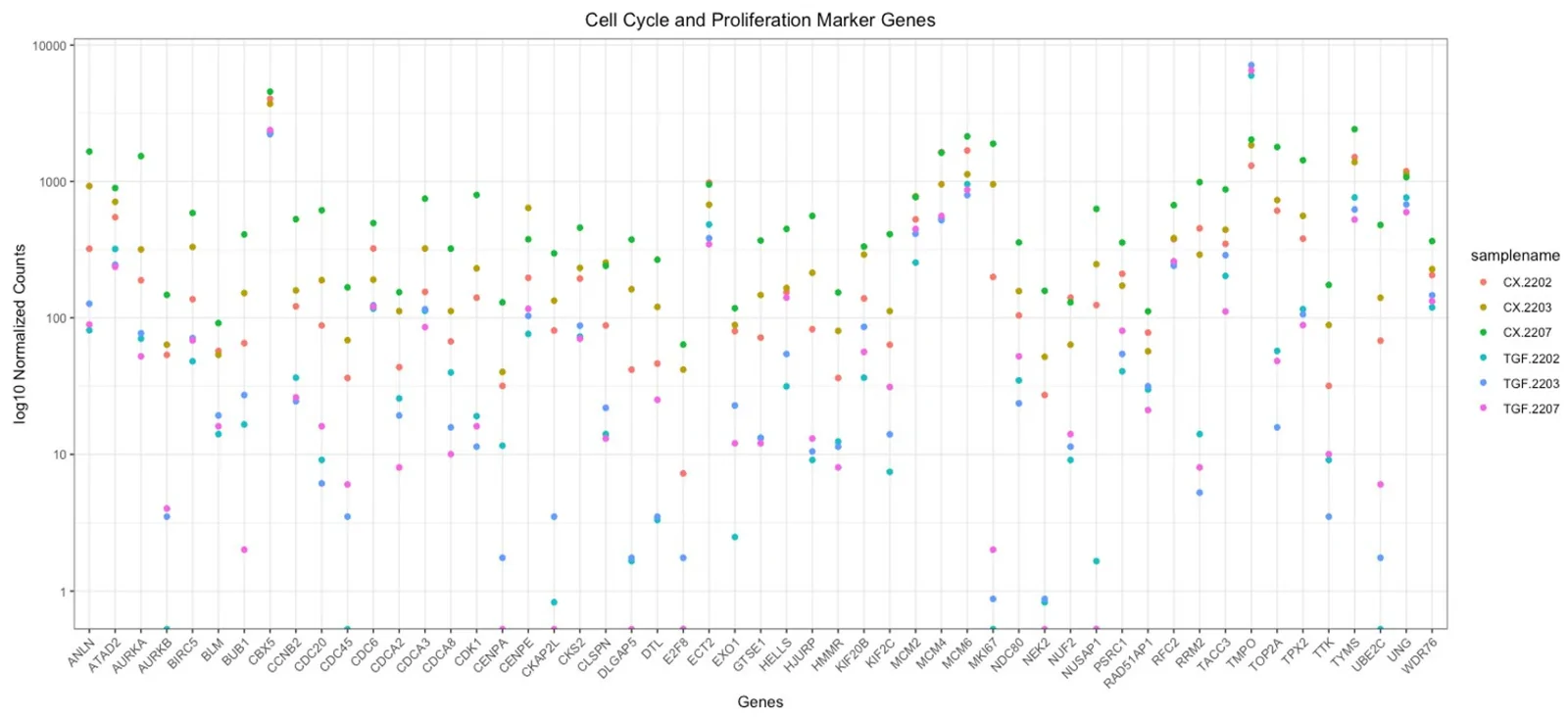
**Cell cycle and proliferation gene expression in HTFs following TGFβ1 treatment.** Dot plot showing cell cycle and proliferation marker genes in control (CX2202, CX2203, CX2207) and treatment groups (TGF2202, TGF2203, TGF2207) with adjusted *p*-value cut-off of 0.01. Many cell cycle and proliferationgenes were downregulated in the treatment group aside from TMPO which was upregulated.

We investigated cell cycle regulatory genes to further understand the role of TGFβ-mediated HTF differentiation or activation. We identified several cell cycle-related gene sets from the Molecular Signatures Database in our dataset [12]. For instances, tumour suppressor genes such as *CDKN2B* were highly upregulated (Figure 2E,F), while *TMPO*, a cell cycle proliferator, was also upregulated in the TGFβ1 treatment group (Figure 3).

### 3.4. Gene Ontology and Functional Analyses

**Figure 4:**
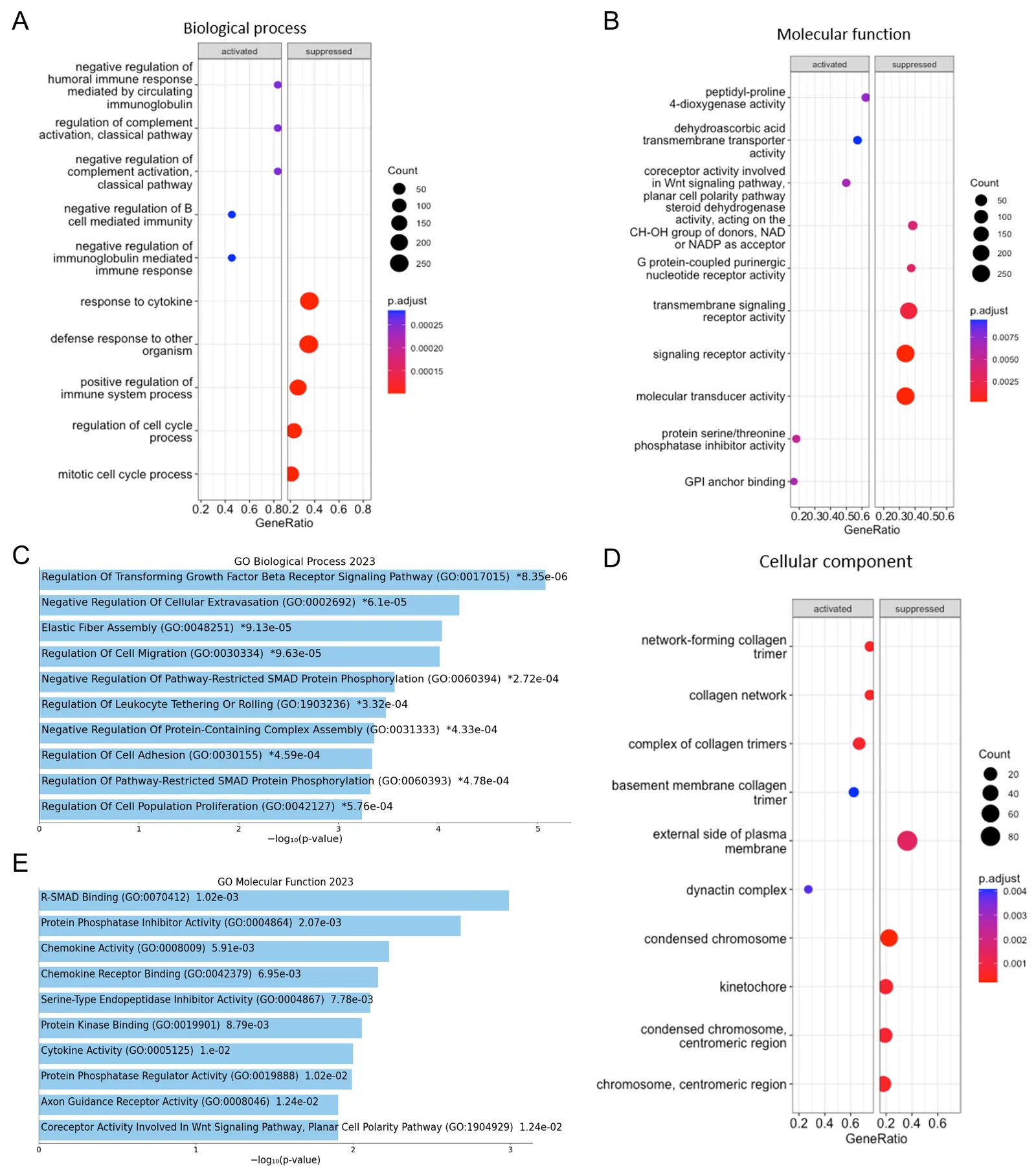
**Gene ontology analysis of HTF following TGFβ1 treatment.** GO and KEGG pathway enrichment analysis of TGFβ1 treatment group compared to control. (**A**) Dotplot shows the activated (upregulated) and suppressed (downregulated) GO terms of biological function associated with all DEGs. The size of the dot is based on gene count enriched in the pathway, and the colour of the dot shows the pathway enrichment significance. (**B**) Dotplot shows the activated (upregulated) and suppressed (downregulated) GO terms of molecular function associated with all DEGs. (**C**) Bar chart of top 10 enriched GO biological functions for top 50 DEGs ordered by *p*-value. (**D**) Dotplot shows the activated (upregulated) and suppressed (downregulated) GO terms of cellular component associated with all DEGs. (**E**) Bar chart of top 10 enriched GO molecular functions for the top 50 DEGs ordered by *p*-value. asterisk (*) indicates the *p*-value.

GO enrichment analysis results revealed that the top significant enrichment pathways were processes involved in fibrosis, including ECM organisation, external encapsulating structure organisation, collagen fibril organisation, and actin filament bundle assembly (Figure 4A–C). Interestingly our results showed that TGFβ1 regulates several signalling pathways such as Smad phosphorylation, the Wnt signalling pathway, and response to cytokines (Figure 4B,C). Regulation of the cell cycle process and regulation of the immune response were highly enriched terms, suggesting that TGFβ signalling controls cell proliferation and survival (Figure 4A). Cellular component terms reveal interesting insight into the molecular processes mediated by TGFβ1. The majority of molecular functions suppressed by TGFβ1 were found to occur within the cell nucleus and be involved in processes including regulation of the cell cycle process and mitotic cell cycle process. Conversely, the activation effects on peptidyl-proline 4-dioxygenase activity (Figure 4A,B) and regulation of collagen occur within the collagen network (Figure 4D,E). These findings suggest that fibroblast to myofibroblast transition is regulated by the ECM microenvironment, and TGFβ1 promotes cell senescence through transcriptional induction of the expression of cell-cycle inhibitors.

**Figure 5:**
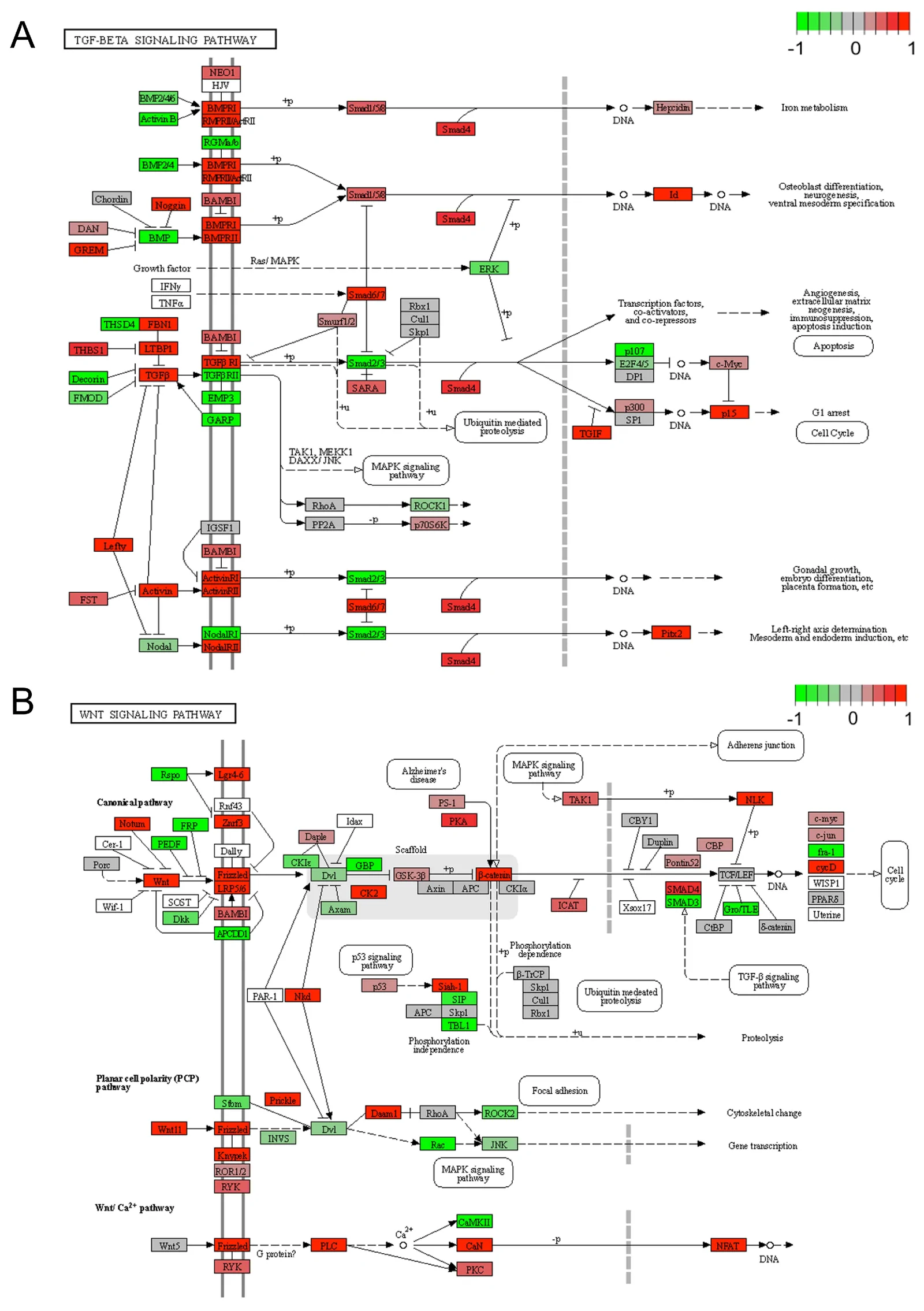
**GSEA of TGF-B and Wnt signalling pathways in HTF following TGFβ1 treatment.** (**A**) KEGG pathway map for TGF-B signalling. (**B**) KEGG pathway map for Wnt signalling pathway. Rectangles correspond to genes or enzymes plotted by fold changes; red = increased fold change, green = decreased, and grey = not available. Colour key gives a range from −1 to 1 and values beyond that range are converted to closest extreme, e.g., values > 1 converted to 1.

**Figure 6:**
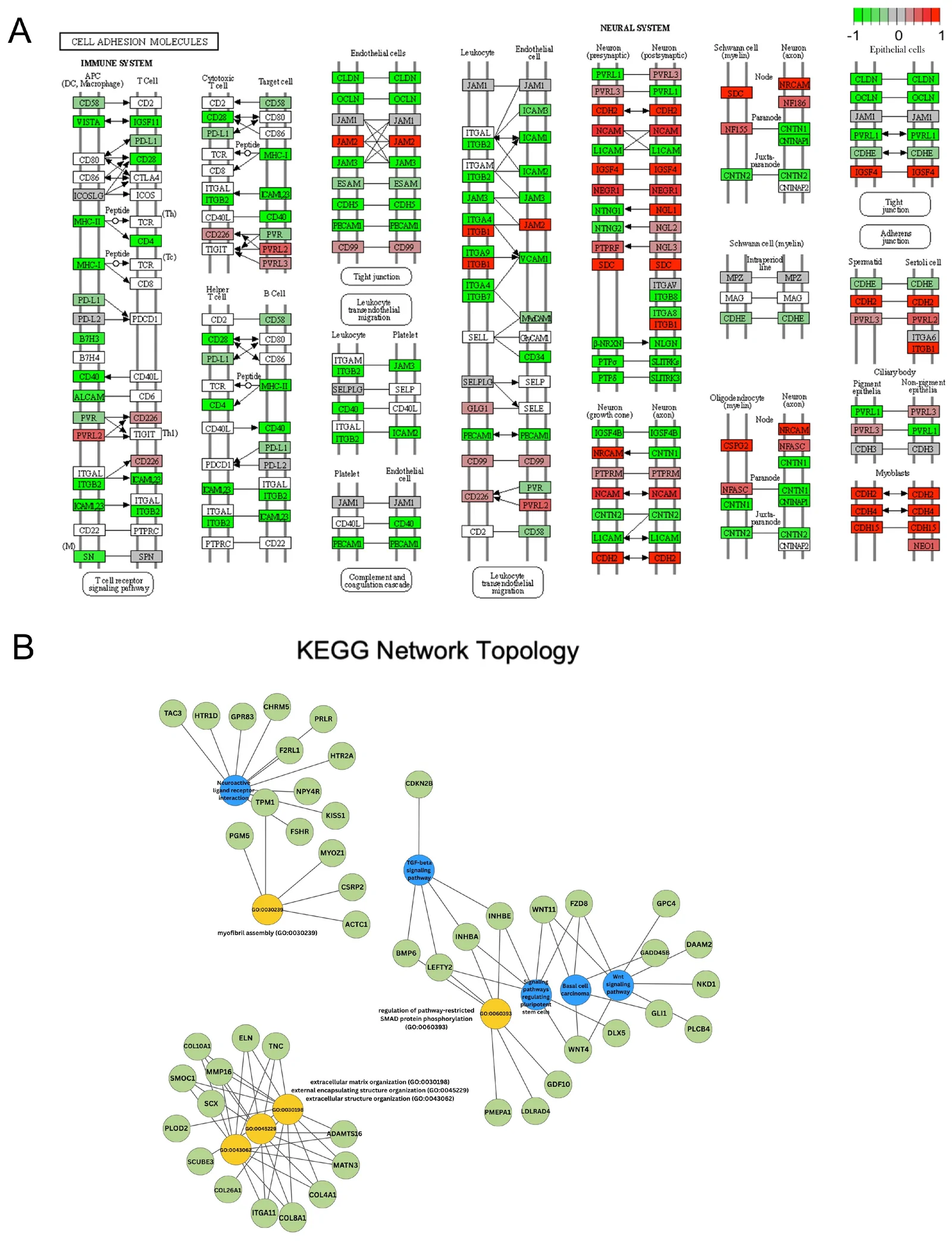
**GSEA of cell adhesion pathway and KEGG network topology in HTF following TGFβ1 treatment.** (**A**) KEGG pathway map for cellular adhesion molecule pathway. Rectangles correspond to genes or enzymes plotted by fold changes; red = increased fold change, green = decreased, and grey = not available. Colour key gives a range from −1 to 1 and values beyond that range are converted to closest extreme, e.g., values > 1 converted to 1. (**B**) Network topology of the top 200 upregulated DE genes to KEGG pathways.

KEGG enrichment analyses revealed that the top enrichment pathways included the TGFβ-signalling pathway, Wnt signalling pathway, cell adhesion molecules and NF-kappa B signalling pathway. BMP, LTBP1, Smad Anchor for Receptor Activation (SARA) and *SMAD4* were shown to play important roles in the TGFβ-signalling pathway (Figure 5A). Genes from the Frizzled and Smad families were found to be involved in the Wnt-signalling pathway. *SMAD3* and *SMAD4* specifically were found to play a role in TGFβ- and Wnt-pathway-mediated effect on cell cycle (Figure 5B). There was significant upregulation of myoblast cell adhesion molecules predominantly driven by Cadherin-2 (*CDH2*) (Figure 6A). Cadherin expression and cell to cell adhesion play a critical role in transition between cellular states. Here we show *CDH2* is potentially a key mediator of intercellular adhesion between myofibroblasts. KEGG network topology showed the crossover between TGFβ-signalling and Smad pathway signalling by *LEFT2*, *BMP6*, and *INHBA* and the interaction between TGFβ-signalling and Wnt-signalling by *INHBA*, *INHBE*, and *FZD8*. As expected, genes encoding for collagen synthesis were responsible for ECM organisation (Figure 6B).

**Figure 7:**
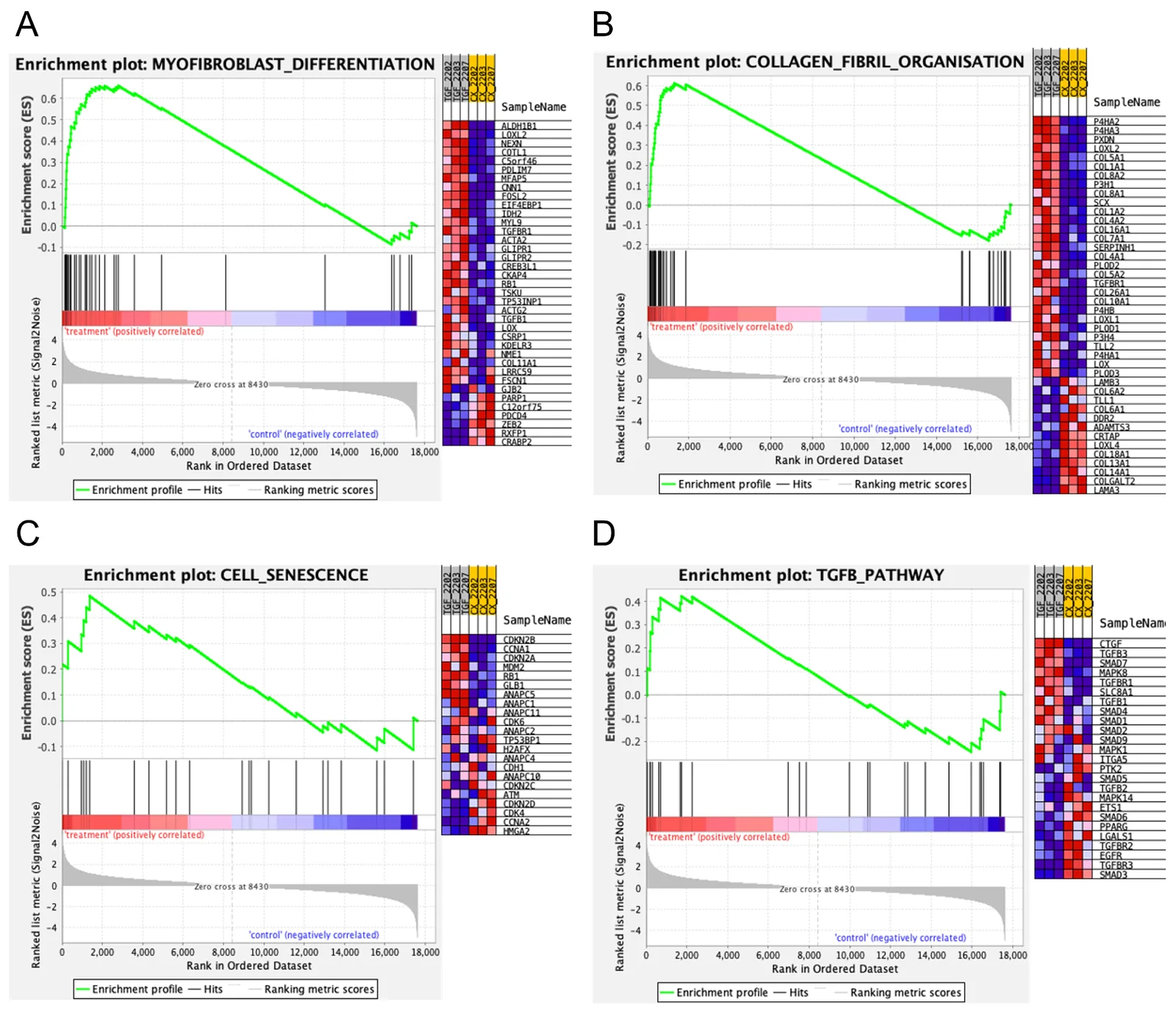
**GSEA analysis in HTF following TGFβ1 treatment.** Gene set enrichment analysis for TGF-B treatment versus control. (**A**) GSEA plot of myofibroblast differentiation with GSEA standard hallmark gene set with heatmap of gene expression; red = upregulated and blue = downregulated. The enrichment score (ES) is indicated. (**B**) GSEA plot for collagen fibril organisation using GSEA standard hallmark gene set. (**C**) GSEA plot for cell senescence using GSEA standard hallmark gene set. (**D**) GSEA plot for TGFB pathway using GSEA standard hallmark gene set.

GSEA was performed to identify top canonical pathways enriched with publicly available KEGG and GSEA-hallmark gene sets. Myofibroblast differentiation had the highest normalised enrichment score, with the most enriched genes being *ALDH1B1*, *LOXL2*, *NEXN*, *COTL1*, and *C5orf46* (Figure 7A, Supplementary Table S1). Genes encoding for collagen synthesis including *P4HA2* and *P4HA3* were enriched in the collagen fibril organisation enrichment plot (Figure 7B, Supplementary Table S1). Tumour suppressors such as *CDKN2B* and oncogenes such as *CCNA1* were highly enriched in cell senescence (Figure 7C, Supplementary Table S1). *CTGF*, *TGFβ1*, *TGFβ3*, and *SMAD7* were highly enriched in the TGFβ pathway (Figure 7D, Supplementary Table S1). *MYOZ1* was found to be significantly enriched in wound healing organisation (Supplementary Figure S3a), and *FBLN5* was significantly enriched in extracellular matrix interaction cell adhesion and within the top 20 significant DEGs (Supplementary Figure S3b). Families of Matrix Metalloproteinases (MMP) and collagen genes were highly enriched in ECM organisation (Supplementary Figure S3c). Altogether, our results identified the genes involved in the myofibroblast transition of HTFs.

## 4. Discussion

This study presents the first RNA-seq study of HTF to myofibroblast activation, a critical TGFβ-dependent process involved in postoperative wound healing following GFS. TGFβ signalling notably plays a critical role in maintenance of tissue homeostasis and is hence highly regulated at several steps in both intracellular and extracellular microenvironments [13]. As this analysis was conducted in myofibroblasts at a 5-day time-point post TGFβ1 treatment, where HTFs expressed a myofibroblast phenotype, we expected our findings to reflect the direct and indirect effects of TGFβ1 on activation and inhibitory signalling in myofibroblasts. Our results highlighted numerous DE genes with known biological roles that potentially contribute to myofibroblast differentiation.

Growth factors and reactive oxidants are released from damaged cells and trigger the activation and proliferation of fibroblasts [14]. *CTGF* is one such growth factor that has been found to play an important role in TGFβ-dependent myofibroblast differentiation and ECM production, indicated by its upregulated expression being highly enriched in the TGFβ pathway (Figure 7D). As an important downstream mediator of TGFβ, *CTGF* has been found to influence myofibroblast differentiation in orbital fibroblasts through increasing fibronectin and αSMA protein expression and has been suggested to provide a safer target for suppression therapy compared to TGFβ [15]. *ACTA2* appears to be important in myofibroblast contraction and migration. *ACTA2* is involved in the regulation of multiple genes relevant to fibrosis, including *COL1*, *GFAP*, *TIMP1*, *TGFβ*, and *ET1* [16]. We found that *NOX4*, a gene encoding for ROS NADPH oxidase 4 enzyme, was upregulated by TGFβ1. NOX4-dependent generation of hydrogen peroxide is a requirement for TGFβ-induced myofibroblast differentiation, as well as ECM production [17]. *TXNDC5* facilitates proper protein folding in the endoplasmic reticulum and mediates redox reactions via interacting with NADPH oxidase [14]. These genes promote conjunctival fibrosis by activation of SMAD3-dependent TGFβ-signalling and lead to differentiation of HTFs into myofibroblasts, resulting in considerable myofibroblast proliferation and ECM production. Notably, we observed the downregulated expression of *SOD3* in our TGFβ-treated samples. As known ROS scavengers, SODs act to reduce oxidative stress in both extra- and intra-cellular environments, with *SOD3* specifically acting in the ECM where it has been found to reduce intracellular ROS levels [18]. Hence, our findings are consistent with *SOD3* deficiency contributing to liver fibrogenesis and TGFβ1-mediated epithelial–mesenchymal transition [19].

TGFβ utilises components of the ECM such as fibronectin and integrins to communicate and control cell behaviour leading to myofibroblast differentiation. *EDN1*, *FN1*, *TNC* and *ITGA11* were upregulated by TGFβ1 and have been reported in myofibroblast differentiation. *EDN1* has been shown to induce resistance to apoptosis in fibroblasts and contribute to fibrogenesis by the abnormal persistence of the myofibroblast phenotype [20]. TGFβ1 can induce expression of fibronectin 1 extra domain A (FN1 EDA) in fibroblasts. *FN1* is not expressed in healthy tissue but is expressed during wound healing; fibroblasts detect FN1 EDA and its presence is required for TGFβ-mediated myofibroblast formation [21]. *TNC* encodes for tenascin-C, an ECM glycoprotein that has been shown to engage integrins to elicit cell-specific responses, such as fibrotic responses, including collagen synthesis and differentiation of myofibroblasts [22]. *TNC* was upregulated in the TGFβ1 sample and has been shown to induce myofibroblast differentiation and migration [23]. Integrins are the main cell-adhesion transmembrane receptors and bind proteins in the ECM such as fibronectin and transduce biochemical and mechanical signals [24]. *ITGA11* was the most significantly upregulated integrin in our data and has been shown to co-localise with alpha-smooth muscle actin-positive myofibroblasts and was correlatively induced with increasing fibrogenesis in human fibrotic organs. *ITGA11* knockdown has been shown to markedly reduce TGFβ-induced differentiation and fibrotic parameters [25]. Our findings are consistent with other studies that reported TGFβ upregulation of *ITGA11* and *CTGF* predominantly binding *ITGA11* [26]. Our results suggest that *ITGA11* is a potential regulator of myofibroblast differentiation in HTF and this association may hold potential as a therapeutic target.

The regulation of cell cycle process and cell senescence pathways were significantly enriched and largely driven by genes *CDKN2*, *CCNA1*, *CDKN2A*, *CDKN2D*, and *CDK4*. TGFβ causes G1-phase cell cycle arrest, and CDKN2B complexes with CDK4 to inhibit its activation, thereby inducingcell cycle arrest [27]. These genes are known cell cycle regulators, and CDKN2b/p15 has been reported to take part in multiple pathologies such as primary open angle glaucoma and cardiac fibrosis [27]. Interestingly, TGFβ1 treatment resulted in downregulation of many cell cycle and proliferation marker genes (Figure 3). The only exceptions to this observation were the upregulated expressions of both the tumour suppressor gene *CDKN2B* (Figure 2E,F) and the cell cycle proliferator gene *TMPO* (Figure 3). Knockdown of *TMPO* in glioblastomas has been found to inhibit apoptosis-dependent cell proliferation and arrest cell cycle progression at the G2/M Phase [28]. Collectively these findings are consistent with other studies reporting the role of TGFβ as a tumour suppressor and inhibitor of cell proliferation, such that cell cycle arrest and apoptosis evasion occur [29].

*TGFβ1* and *TGFβ3* were upregulated in the TGFβ1 treatment group. The three TGFβ isoforms have highly homologous receptor-binding domains and similar effects on target cells but with divergent primary amino acid sequences in the latency-associated peptide domains. Isoform-specific function may be related to different expression in cell types and different extracellular environments [30]. *TGFβ1* was enriched in pathways such as the TGFβ pathway, myofibroblast differentiation, and collagen fibril organisation, whereas *TGFβ2* and *TGFβ3* were not. This suggests that HTF-induced fibrosis is largely a *TGFβ1*-driven process. *TGFβ3* appears to play a role in regulation of epithelial to mesenchymal transition and the TGFβ pathway rather than myofibroblast differentiation, which is consistent with previous reports of TGFβ3 expression being restricted to mesenchymal cells [30].

Our findings highlight the important molecular mechanisms of TGFβ signalling, particularly positive and negative regulation of pathway-restricted Smad protein phosphorylation (Figure 4C; GO:0060393 & GO:0060394). The main genes upregulated in this biological process were *GDF10*, *PMEPA1*, *LDLRAD4*, *INHBA*, *LEFTY2*, *BMP6* and *SMAD7*. Canonically, TGFβ signal transduction occurs via activation of downstream mediators Smad2 and Smad3, which are negatively regulated by Smad7 as part of a negative feedback loop [31]. Smad7 inhibits phosphorylation of R-Smads(2/3) by competitively binding TβR-I activated by TβR-II [32]. We speculate that the upregulated expression of *SMAD7* is associated with the upregulated expression and enrichment of the *SMURF1* gene as their encoded proteins regulate each other’s functions. For Smad7 to perform its inhibitory function it is required to interact with Smurf1 to be exported from the nucleus to the cytoplasm [33]. *INHBA* encodes inhibin beta-A, which functions as a subunit of activin A and is classified as a ligand of the TGFβ superfamily [34]. *INHBA* attenuation in renal fibroblasts has been found to significantly reduce their expression of profibrotic markers, inhibiting cell migration and proliferation [35]. To promote the TGFβ-signalling pathway, *INHBA* is able to homodimerize and bind the activin type I/II receptor complex, which then can phosphorylate R-Smad proteins [36,37]. Hence, we suspect that upregulated *INHBA* expression correlates with upregulated expression of *ACVR1* (encodes type I receptor, ALK2) and *ACVR2B* (encodes type II receptor) to promote Smad-dependent TGFβ signalling.

While the results achieved in this study advance our understanding of the molecular mechanisms of ocular fibrosis following GFS, this study has its limitations. Although the current literature supports RNA-seq analysis of HTF as a homogenous population, single-cell RNA-seq would potentially identify heterogeneity or subtypes within the HTF and myofibroblast population. Another limitation of the study was that specimen collection was performed at the time of GFS, from patients who were receiving medical therapy for glaucoma (Supplementary Table S2). The effects of these medications on gene expression remain unknown, although exposure to the preservative benzalkonium chloride is associated with adverse effects including trabecular meshwork apoptosis, conjunctival and anterior chamber inflammation, and corneal cytotoxicity [38,39,40,41]. Also, this study utilised three biological replicate samples, and future studies to increase sample size would be important to improve the statistical power to distinguish universal or patient-specific effects. While this study highlighted the transcriptomic changes following TGFβ-induced myofibroblast differentiation in HTF and revealed numerous DE genes involved in the process, it remains unclear whether these DE genes are drivers or consequences of myofibroblast differentiation. Future studies to experimentally validate these DE genes and understand their functions in HTFs would be of high interest, providing new insights into their roles in myofibroblast differentiation. These functional studies could reveal useful information that potentially lead to identification and prioritisation of novel anti-fibrosis drug targets. Finally, this study utilised primary HTF isolated from patients following GFS. While this provided a clinically relevant ex vivo model to study myofibroblast transition, this ex vivo model does not recapitulate the complex cellular environment in conjunctival fibrosis, such as the inflammatory responses at the surgery site. To bridge this translational gap, future drug development studies should utilise in vivo models, such as the mouse GFS model [42] or the rabbit GFS model [43]. Also, an in vitro model using complex engineered tissue of the conjunctiva and Tenon’s capsule [44] would be useful to test local drug delivery in the subconjunctival tissue.

In investigating a model of HTF transition into myofibroblasts at the transcriptomic level, we gained greater insight into the novel molecular interactions and causal pathways of ocular fibrosis. This study establishes a gene signature of myofibroblast activation from HTFs that is uniquely characterised by genes involved in regulation of myofibroblast differentiation, collagen fibril organisation, cell cycle arrest, TGFβ-signalling pathways, and wound healing organisation. The results of this study establish an essential milestone for the future development of effective antifibrotic therapies targeting scar tissue formation following GFS.

## Data Availability

The transcriptome data generated in this study are available in the NCBI Gene Expression Omnibus database (accession GSE275110), including raw data and processed data.

## Supplementary Materials

The following supporting information can be downloaded at https://www.mdpi.com/article/10.3390/biomedicines14081718/s1: Supplementary Figure S1: Characterisation of Human Tenon’s fibroblasts (HTFs) Cultured primary HTFs were stained with S100A4 (red) and nuclei counterstained with DAPI (blue); Supplementary Figure S2: Time-Point Study Triplicate Phenotypic Imaging of HTFs after 3–5 days of TGFβ Treatment Cultured primary HTFs were stained with αSMA-antibody and nuclei counterstained with DAPI (blue); Supplementary Figure S3: GSEA for TGF-B treatment versus control; Supplementary Table S1: GSEA table for TGF-B1 treatment compared to control GSEA enrichment results for the top 10 enrichment terms; Supplementary Table S2: Prescribed glaucoma medications used by patients. Supplementary Data S1: DEG analysis of HTF following TGFβ1 treatment.
