# Supplementary material for: Transcriptomic Study of Mechanism Driving Conjunctival Fibrosis Following Glaucoma Filtration Surgery
Source: Biomedicines. 2026 Jul 31;14(8):1718. doi: 10.3390/biomedicines14081718 (PMC13510143; doi:10.3390/biomedicines14081718)
Supplement: Supplementary file 1 [file biomedicines-14-01718-s001.zip › Pasvanis 2026 transcriptomic study of conjunctival fibrosis suppl info.pdf]

Supplementary Information

Transcriptomic Study of Mechanism Driving Conjunctival Fibrosis Following Glaucoma Filtration Surgery

Author information
Zoe Pasvanis, Antony Boynes, Roy C. K. Kong, Elsa C. Chan, Raymond C. B. Wong, Jennifer Fan Gaskin

Supplementary figures

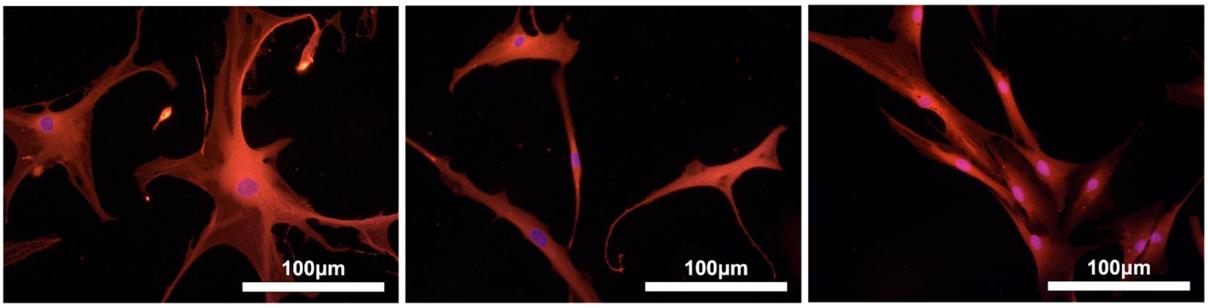

Supplementary Figure S1: Characterisation of Human Tenon's fibroblasts (HTFs) Cultured primary HTFs were stained with S100A4 (red) and nuclei counterstained with DAPI (blue). Fibroblast phenotype verified by cells presenting red-stained cytoplasm. (N=3).

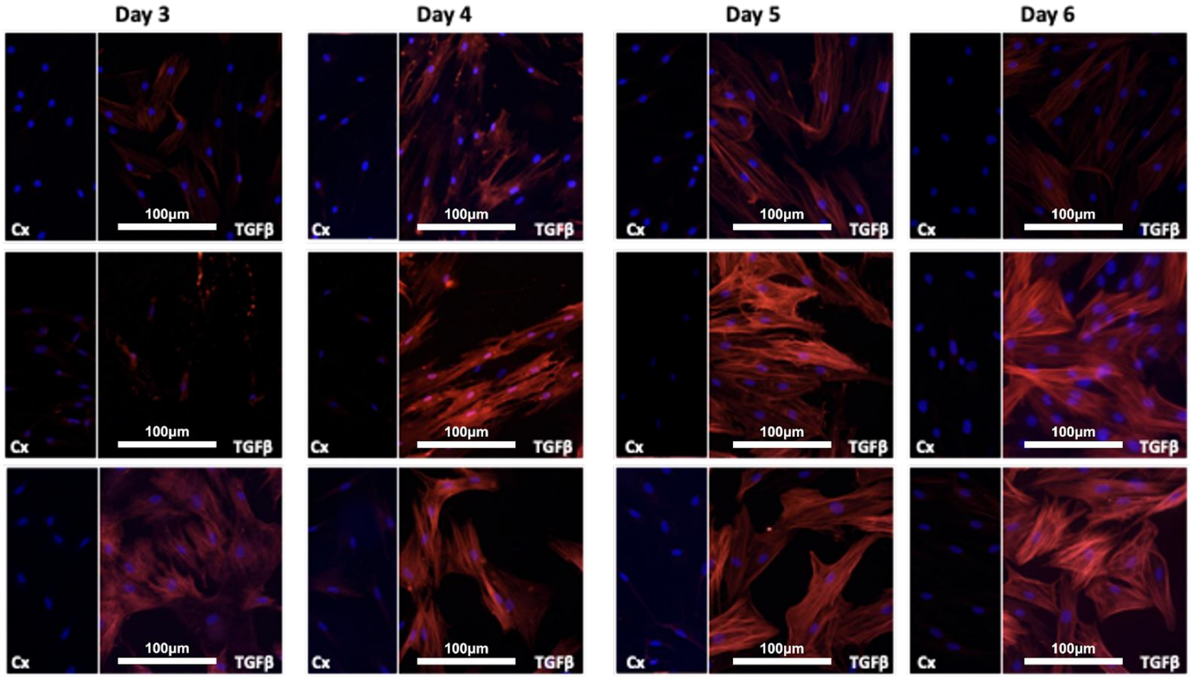

Supplementary Figure S2: Time-Point Study Triplicate Phenotypic Imaging of HTFs after 3-5 days of TGFβ Treatment Cultured primary HTFs were stained with αSMA-antibody and nuclei counterstained with DAPI (blue). Red-stained fibres indicate de novo expression of αSMA in collagen stress fibres of myofibroblasts. Cx= Control sample; TGFβ = Transforming Growth Factor Beta treated sample (N=3).

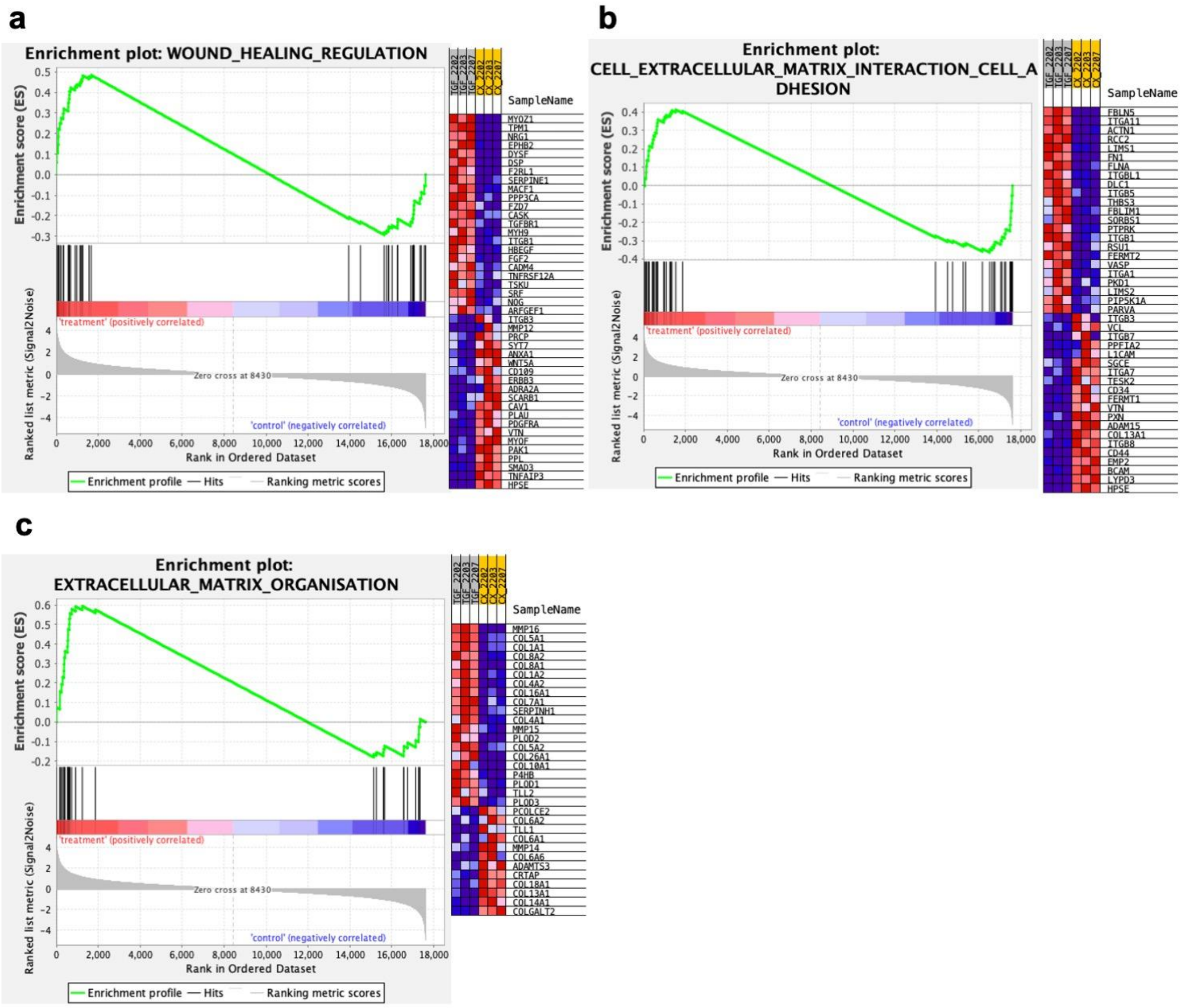

Supplementary Figure S3: GSEA for TGF-B treatment versus control (a) GSEA plot of wound healing regulation with GSEA standard hallmark gene set with heatmap of gene expression red = upregulated, and blue = downregulated. The enrichment score (ES) is indicated. **(b)** GSEA plot for cell extracellular matrix interaction cell adhesion using GSEA standard hallmark gene set. **(c)** GSEA plot for extracellular matrix organization using GSEA standard hallmark gene set.

Supplementary Table S1: GSEA table for TGF-B1 treatment compared to control GSEA enrichment results for the top 10 enrichment terms. ES: enrichment score, NES: normalized enrichment score, NOM p-value: nominal p-value, FDR q-value: false-discovery rate q-value, FWER p-value: familywise-error rate p-value.

GS DETAILS from MSigDB	Size	ES	NES	NOM p-value	FDR q-value	FWER p-value	Rank at max
1. MYOFIBROBLAST DIFFERENTIATION	37	0.66	2.27	0.000	0.000	0.000	2800
2. COLLAGEN FIBRIL ORGANISATION	42	0.61	2.12	0.000	0.000	0.000	1310
3. EXTRACELLULAR MATRIX ORGANISATION	32	0.59	1.93	0.000	0.001	0.003	1242
4. WOUND HEALING REGULATION	43	0.48	1.74	0.000	0.016	0.051	1669
5. ER STRESS UPR GENE SETS	43	0.48	1.69	0.014	0.021	0.083	1610
6. REGULATION EPITHELIAL TO MESENCHYMAL TRANSITION	24	0.50	1.57	0.022	0.050	0.220	1550
7. CELL SENESCENCE	22	0.48	1.47	0.060	0.089	0.397	1371
8. CELL EXTRACELLULAR MATRIX INTERACTION CELL ADHESION	43	0.41	1.46	0.036	0.081	0.412	1540
9. TGFB PATHWAY	25	0.42	1.31	0.131	0.163	0.702	1737
10. WNT PATHWAY	18	0.46	1.29	0.163	0.164	0.749	1338

Supplementary Table S2: Prescribed glaucoma medications used by patients.
Glaucoma medications being used by patients at the time of tissue biopsy collection.

Glaucoma medications	Method of application	Preservative	Mechanism of action
Bimatoprost	Topical	Benzalkonium chloride	Prostaglandin analog
Brinzolamide/timolol fixed combination	Topical	Benzalkonium chloride	Carbonic anhydrase inhibitor Β-blocker
Brimonidine	Topical	Sodium chloride	α2-adrenergic agonist
Brinzolamide/brimonidine	Topical	Benzalkonium chloride	Carbonic anhydrase inhibitor α2-adrenergic agonist
Timolol/latanoprost fixed	Topical	Benzalkonium chloride	Β-blocker prostaglandin analog
Prednifrin forte	Topical	Benzalkonium chloride	Glucocorticoid
Fluoromethalone	Topical	Benzalkonium chloride	Corticosteroid
Acetazolamide	Oral	N/A	Internal carbonic anhydrase inhibitor
